# Endothelial protein C receptor expressed by ovarian cancer cells as a possible biomarker of cancer onset

**DOI:** 10.3892/ijo.2012.1492

**Published:** 2012-05-18

**Authors:** ELODIE DUCROS, SHAHSOLTAN MIRSHAHI, DALEL AZZAZENE, SOPHIE CAMILLERI-BROËT, ELIANE MERY, HALEMA AL FARSI, HAMDA ALTHAWADI, SAMAHER BESBESS, JEAN CHIDIAC, ERIC PUJADE-LAURAINE, AMU THERWATH, JEANNETTE SORIA, MASSOUD MIRSHAHI

**Affiliations:** 1 National Institute for Medical Research (INSERM), Cordeliers Research Center (UMRS 872), University of Pierre and Marie Curie, Sorbonne University (UPMC) and University of Paris Descartes, Paris;; 2 Diagnostica Stago, Gennevilliers;; 3 Claudius Regaud Institute, Toulouse;; 4 Hôtel-Dieu Hospital, Paris, France

**Keywords:** endothelial protein C receptor, ovarian cancer, DNA sequence, biomarker, CA125

## Abstract

Coagulation disorders often accompany cancer onset and evolution, which, if not properly managed, could have grave consequences. Endothelial protein C is an important regulator of homeostasis and acts through its high affinity binding to its transmembrane receptor (EPCR). Soluble (sEPCR) which results from the proteolytic cleavage of the membrane bound form can trap activated endothelial protein C and deprive it of its anti-coagulant function. In this study, the expression of EPCR and its soluble form (sEPCR) released into plasma as a result of proteolytic cleavage were investigated in ovarian, breast, lung and colorectal cancer biopsies, as well as in ascitic cell clusters and peritoneal fluid from ovarian cancer samples. In parallel, breast, ovarian, lung and colorectal cancer cell lines were investigated for the expression of EPCR. The integrity of the EPCR gene sequence as well gene haplotypes were ascertained in the established cancer cell lines in order to understand their eventual regulatory functions. The results from the present study indicate that in cancer patients, the levels of sEPCR are significantly higher than the normal range compared to healthy volunteers. The increase in the levels of sEPCR parallels the increase in CA125, showing a close correlation. Therefore, the detection of sEPCR in cancer and during the post-treatment period could be taken into account as an additional marker that could re-inforce the one obtained using CA125 alone as a marker of cancer cell mass.

## Introduction

The search for newer and more reliable biomarkers is a continuing research endeavour. Biomarkers are crucial in narrowing down on a precise diagnosis and are equally important during the follow-up stage in disease management. Preliminary experiments in our laboratory indicated that endothelial protein C could be a possible biomarker candidate.

The endothelial protein C along with its receptor displays pleiotropic functions. One such recognized function is its role as an important regulator of homeostasis, besides being implicated in the systemic response to acute inflammation. Protein C zymogen binds to endothelial protein C receptor (EPCR) with high affinity and stimulates its activation via the thrombinthrombomodulin complex. Activated protein C (aPC), together with its co-factor, protein S, degrade factors Va and VIIIa and thereby interfere with thrombin generation (1–3).

EPCR is a type 1 transmembrane glycoprotein (CD201) that shares considerable homology with the major histocompatibility complex (4). EPCR is known to be constitutively released in the plasma in a free soluble form as a result of proteolytic cleavage. This soluble form of EPCR (sEPCR) has the ability to trap free aPC, thereby depriving it of its anti-coagulant function within the surrounding environment (5,6). It has been shown that the shedding of EPCR by human umbilical cord endothelial cells (HUVECs) is effectively regulated by IL-1β and TNF-α, and downstream by MAP kinase signaling pathways and metalloproteinases (7).

During acute inflammation, a significant increase in circulating sEPCR is observed which in turn could provide an early biological marker of sepsis outcome (8). It is known that cytokines, such as IL1-β, and TNF-α, as well as endotoxins, can attenuate the expression of EPCR which then leads to diminution in plasma aPC (9). However, the nature of the anti inflammatory action of aPC remains unclear.

The EPCR gene carries 13 single nucleotide polymorphisms, which define 3 haplotypes. One of these haplotypes, A3, encodes a protein, which is more sensitive than the other two to the action of shedding enzymes, which can result in a marked increase in the level of sEPCR. The presence of the A3 haplotype therefore coincides with the presence of a high sEPCR level, the latter being a candidate risk factor for venous thrombosis (10–12). Interest in EPCR/aPC in relation to tumor biology is gaining momentum with the appearance of an increasing number of publications (13–16).

The appearance of high levels of sEPCR in malignant tumors during disease and during relapse could have an implication in venous thrombosis and if detected early enough could serve as a useful indicator and therefore, preventive measures could be taken. Taking this evidence into consideration, we undertook a detailed study of aPC/EPCR not only in a large cohort of tumor biopsies, patient ascitic fluid and plasma, but we also extended our study to include *in vitro* cultured tumor cell lines.

## Materials and methods

### 

#### Reagents

Reagents were obtained from the following sources: primary antibody AF2245 against EPCR (R&D Systems, Minneapolis, MN, USA); primary antibody ATAP2 against PAR-1 (Invitrogen, Carlsbad, CA, USA); biotinylated anti-rabbit, anti-mouse and anti-goat IgG, streptavidin-fluorescein conjugate (Amersham, Buckinghamshire, UK); rabbit anti-goat HRP (DakoCytomation, Glostrup, Denmark); phycoerythrin-coupled anti-P-gp antibody (Millipore, Billerica, MA, USA); human recombinant aPC (Lilly, Suresnes, France); U0126 and wortmannin (Calbiochem, San Diego, CA, USA). The recombinant form of human aPC marketed as Xigris, was obtained from Eli Lilly (Indianapolis, IN, USA). The PCR primers were designed with the Primer3 program, BLAST verified, and synthesized by Eurobio (Les Ulis, France).

#### Cells

The human cancer cell lines used were: ovarian (OVCAR, ATCC), breast (MDA-MB231 ATCC), lung (A549) and colorectal (HT-29, HCT-8R ATCC). Cells were cultured in RPMI-1640 medium containing 10% fetal calf serum, penicillin (50 U/ml), and streptomycin (50 *μ*g/ml) and incubated in a humidified atmosphere containing 5% CO_2_ at 37°C, as recommended by the supplier (PAA Laboratories Inc., Etobicoke, ON, USA).

#### Conditioned medium

Cells, seeded in two 25-cm^2^ flasks, were grown to 80% confluency and then incubated in 1 ml serum-free culture medium. aPC (10 *μ*g/ml) was added to one flask, while the second one, without aPC, served as the control. Cells were pelleted by centrifugation after 18 h and the conditioned medium was collected and aliquoted.

### Plasma, ascites and mononuclear cells

#### Plasma samples

Blood (n=79) samples from patients with ovarian cancer were obtained from the Oncology Department of Hôtel-Dieu Hospital (Paris, France) after informed consent, in accordance with the rules of the revised Helsinki protocol. A total of 79 patients with an age range of 39–90 years (mean ± SD, 62±14 years) were selected. Patients received anti-coagulants.

#### Ascitic cells

Peritoneal fluid from 23 cancer patients of the Hospital Hôtel-Dieu was collected. As ascite evacuation is part of the routine management of patients, only oral consent was obtained from them. Cells from ascitic fluid were pelleted by a short spin at 1,000 rpm and the supernatant was collected.

#### EPCR-specific antibody production

Three EPCR-specific peptides were chosen based on information provided by the crystallographic structure (21) and with the aid of the Cn3D 4.1 program for the tridimensional structure of EPCR. The EPCR-specific peptides synthesized were as follows: peptide no. 1, 51-GGHLT HVLEG PDTNT TIIQL-70; peptide no. 2, 68-IQLQP LQEPE SWART QSGLQ-87; and peptide no. 3, 157-TSGVV TFTLQ QLNAY NRTRY-176. One of the peptides (peptide no. 3) contained three essential residues required for aPC binding (Q166, R173 and T174). The peptides were KLH-coupled and injected in combination with Freund’s adjuvant every two weeks into New Zealand white rabbits (150 to 300 *μ*g per injection) provided on contract by Agro-Bio (La Ferté St. Aubin, France), followed by immunoglobulin purification. The fifth and last antigen injection was on the 56th day and sera from rabbits were collected on the 77th day. The specifity of polyclonal antibodies to peptides was tested using a competition assay by the ELISA method using purified peptides.

#### Immunolabeling

The presence of EPCR proteins in cancer cells was revealed by immunocytochemistry as previously described (17). As the controls, isotypic antibodies were used in parallel and the nuclei were DAPI-labeled. FACS analysis was performed on cells detached by accutase (PAA) treatment and immunolabeled as previously described (17) with EPCR (20 *μ*g/ml) antibody and compared with the isotypic control. Ascitic cells were examined as previously described (17). Cells were observed after staining with methylene blue and eosin.

#### Tissue microarray (TMA)

EPCR expression was examined on 4 mm-thick routinely processed paraffin sections of tumor biopsies as described by Rafii *et al*(18). Four different TMAs were prepared. Ovarian cancer TMA contained 146 samples (before treatment, n=84 and after treatment, n=62), breast tumor TMA 120 samples, while colon cancer TMA had 30 samples and the lung cancer TMA had 24. EPCR proteins were revealed by fluorescent immunolabeling and compared to the controls using isotypic antibodies.

#### sEPCR-ELISA assay

sEPCR in cultured cell supernatants and in ascitic fluid was measured using Asserachrom sEPCR immunoassay as recommended by the commercial supplier (Diagnostica Stago, Parsippany, NJ, USA).

#### Rerverse transcription-polymerase chain reaction (RT-PCR) analysis

The cancer cell RNA extracts were prepared using the Nucleospin RNA-II kit (Macherey-Nagel EURL, Hoerdt, France). Following reverse transcription [Mu-MLV reverse transcriptase and oligo(dT) primers], PCR was performed with TaqDNA polymerase (Gibco-BRL, Paisley, UK). Specific primers for EPCR synthesis were as follows: sense, 5′-CAA CTT CAG GAT GTT GAC AA-3′; antisense, 5′-CTA CAG CCA CAC CAG CAA T-3′ to yield a product size of 692 bp (18). The PCR products, along with a 100-bp DNA ladder, were analyzed by electrophoresis on agarose gels containing ethidium bromide.

#### Gene sequencing

The cancer cell RNA extracts were prepared and subjected to reverse transcription as described above. Cancer cell DNA extracts were prepared using the Nucleospin Tissue kit (Macherey-Nagel EURL). Genomic DNA and cDNA samples were shipped on dry ice to the Qiagen Sequencing Service. Samples were amplified with HotStarTaq Plus DNA Polymerase using custom-made primers, designed by Qiagen. These primers were selected according to the EPCR sequence available under GenBank accession nos. AF106202 or BC01445. Reference AF106202 includes the entire gene and promoter region (8,167 bp) whereas BC01445 represents the mRNA (1,381 bp). Both strands of DNA were sequenced with BigDye 3.1 Terminator Chemistry (Applied Biosystems, Carlsbad, CA, USA), using the ABI Sequence Analyzer 3730XL. The genomic DNA sequence was aligned with the GenBank reference sequence AF106202 whereas the cDNA sequenced was aligned with BC01445.

## Results

### 

#### Detection of protein C receptor in situ in ovarian cancer

**Figure 1:**
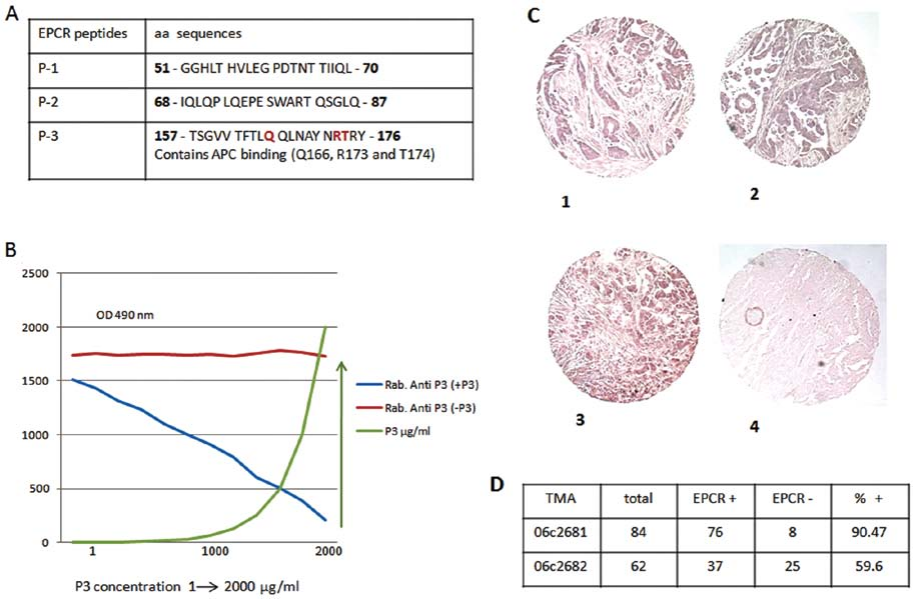
Preparation of anti-EPCR antibodies for *in situ* detection of EPCR in ovarian cancer biopsies. (A) The three EPCR-specific peptides (P1, P2 and P3) were chosen based on information provided by the crystallographic structure and synthesized. (B) The specificity of the polyclonal antibodies to these peptides were tested by ELISA using a competition assay and purified peptides. (C) *In situ* detection of EPCR performed using immunohistochemistry of EPCR on ovarian cancer tissue microarray. (C-1 and -2) EPCR was revealed either by standard peroxydase staining befor treatement and (C-3) after treatement. (C-4) Negative control, performed with an isotypic antibody (initial magnification, ×20). Results are representative of three independent experiments, each of which gave similar results. The results were analysed and are presented in (D). Rab, rabbit.

**Figure 2:**
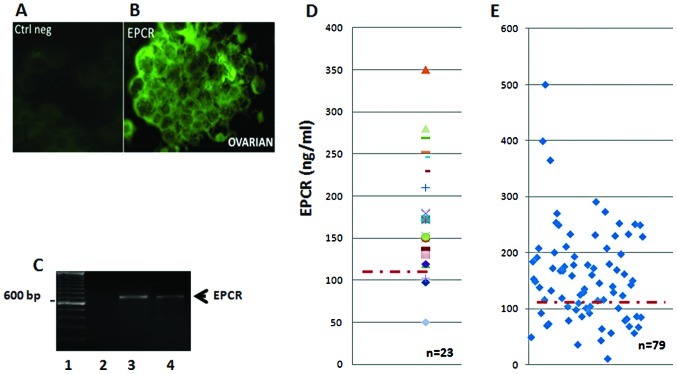
Immunodetection of EPCR in ascitic cell clusters, ascitic fluid and plasma from ovarian cancer patients. (A) Isotype control monoclonal anti-EPCR antibody used for the detection of cell-associated EPCR in ovarian cancer cell clusters from ascites (B). These results were confirmed by RT-PCR using specific primers for EPCR in several patients. Lanes 3 and 4 show results obtained for two distinct patients. Lane 1 shows the DNA ladder used as marker of fragment size and lane 2 represents the negative control. The expected 692-bp fragment was observed as shown in lane 4. The amount of sEPCR in (D) 23 samples from ascitic fluid and (E) 79 plasma samples from ovarian cancer patients was quantified by ELISA.

**Figure 3:**
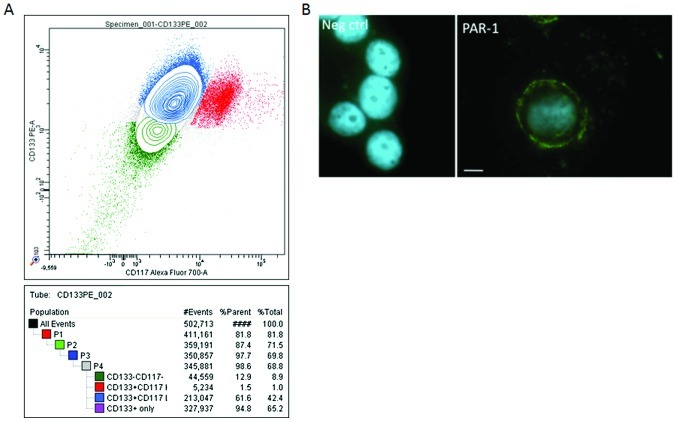
Immunoanalysis of the ovarian cancer cell line, OVCAR-3. (A) Three subpopulations of OVCAR cells were identified by the cell sorter. They were CD133^+^ (65.2%), CD133^+^/CD117^+^ (42.4%) and CD133^−^ (8.9%). (B) PAR-1 immunocytochemistry. OVCAR cells were grown on glass bottom chamber slides, fixed and successively incubated with PAR-1 antibody, appropriate biotinylated secondary antibody and FITC-streptavidin. Isotype antidodies were used in parallel and the nuclei were DAPI-labeled. Initial magnification, ×400. Scale bar represents 10 *μ*m.

**Figure 4:**
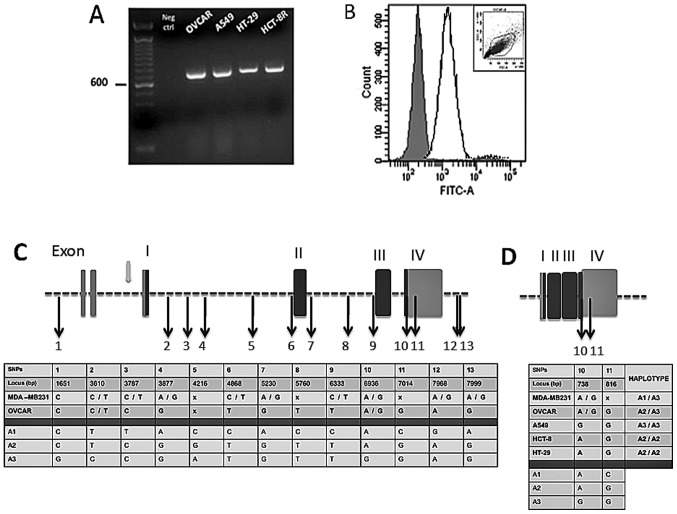
EPCR gene sequence analysis from ovarian, breast, lung and colorectal cancer cell lines. (A) Gel electrophoresis of EPCR (RT-PCR products) of ovarian (OVCAR), lung (A549), colorectal (HT-29) and ileocecal (HCT-8R) cancer cell lines. Expected bands of 692 bp were observed as shown and the control without nucleic acid (Neg ctrl) remained negative. (B) Flow cytometry analysis of EPCR in OVCAR. The solid line shows EPCR staining and the isotype control as represented by the shaded area. The cytogram gate is displayed in the upper corner. (C) Breast and ovarian cancer cell EPCR DNA alignment with GenBank data (reference no. AF106202). (D) Ovarian, breast, lung and colon cancer cell EPCR cDNA alignment with GenBank data (reference no. BC01445). DNA and cDNA extracts were shipped to the Qiagen Sequencing Service. Both DNA strands were sequenced and nucleotides were numbered according to the appropriate GenBank reference no. The 13 single nucleotide polymorphisms (SNPs) initially described in endothelial cells were also found here allowing us to designate 3 haplotypes (A1, A2, A3). Exons are presented as vertical rectangular blocks; darkly shaded regions are the ones that are transcribed. Black arrows indicate the SNPs. Gray arrow shows a promoter region. The ‘x’ indicates positions of bases that the commercial firm, Qiagen, was uncertain of; it can represent any one of the four bases.

Three rabbit anti-EPCR-specific peptides were produced. One of these peptides (peptide 3) contained an aPC binding (Q166, R173 and T174) domain (Fig. 1A). The specificity of these antibodies was tested using a competitive immune assay based on the binding competition between rabbit anti-peptides and the corresponding peptide. Results are presented only for peptide 3 (Fig. 1B). These antibodies were used for the immune analysis of EPCR in the present study. EPCR expression *in situ* was evaluated in various cancer biopsies by TMA using anti-peptide 3 antibody. As presented in Fig. 1C and D, of the 146 biopsies from ovarian cancer tested before (n=84, Fig. 1C–1 and C–2) and after (n=62, Fig. 1C–3) treatment, 90.47 and 56.6% of biopsies were positive for the protein C receptor, respectively. The control is presented in Fig. 1C–4.

Similarly, EPCR was detected in 20 out of 24 lung cancer biopsies, representing approximately 80% of positive samples in this case. Also, of the 30 colon cancer biopsies tested, 20 (65%) were positive for EPCR expression (data not shown). Therefore, the presence of EPCR in tumors, whatever their origin, seems to be, more or less, common.

#### Evaluation of sEPCR in ascitic cell clusters, fluid samples and plasma from ovarian cancer patients

Twenty-three ascitic fluid samples and their floating cell clusters were also screened for the presence of EPCR (Fig. 2). The data in Fig. 2 indicate the presence of membrane-bound EPCR detected by immunohistochemistry (Fig. 2B), while the isotype control remained negative (Fig. 2A). All ascitic cell clusters were found to be positive for EPCR protein expression. Similar results were also obtained when we used rabbit anti-EPCR peptides as the probe (data not shown). These results were then confirmed using RT-PCR analysis. Cells in the floating aggregates (clusters) were found to transcribe the EPCR gene as an amplified band consistent with the predicted size (Fig. 2C).

The quantity of sEPCR in ascitic supernatants and plasma from ovarian cancer was assessed by ELISA. All samples tested positive, and exhibited a concentration well above the baseline plasma value of 100 ng/ml (Fig. 2D). Ascite samples (91%) tested positive, revealing a concentration noticeably higher than the plasma baseline value estimated at 100 ng/ml. The above cited results correlate with fibrin deposits generally observed in ascites (data not shown). Finally, in plasma samples from ovarian cancers, once again, 70% of the samples revealed a concentration well above the baseline plasma value (Fig. 2E).

#### sEPCR expressed by ovarian cancer cells may be a biomarker of cancer onset

We assessed by ELISA the quantity of sEPCR and detectable CA125 in a relatively small number (n=29) of patients with ovarian cancer before treatment. This allowed us to establish, using the Spearman’s test, a positive correlation between plasma sEPCR and CA125 in the patient population (Spearman’s coefficient, Rho = 0.36).

We divided all patients into two subgroups: group 1 (n=12) with the amount of plasmatic sEPCR below the baseline (68 to 117 ng/ml) and group 2 (n=17) where the concentration of sEPCR was noticeably higher than the plasma baseline value (140 to 250 ng/ml, n=17). The results are presented in Table I.

The median value obtained was 68.5 ng/ml for sEPCR and 197 ng/ml for CA125 in the first group and 198 ng/ml for sEPCR and 339 ng/ml for CA125 in the second group. The results obtained show that in the second group, when CA125 increased by 1.72-fold, the amount of sEPCR also increased by 2.9-fold. Thus, in the second group, there was a positive correlation between sEPCR and CA125, indicating that sEPCR could be measured as an indicator of ovarian cancer along with the currently used measure of CA125.

#### OVCAR-3 ovarian cancer cell line expresses PAR-1 antigens, CD133 and CD117

Analysis by cytometry of OVCAR-3 cells using CD133 and CD117 antibodies revealed a positive immune reactivity with these. The representative experiments presented in Fig. 3A show that 65.2% of OVCAR-3 wild-type cells expressed CD133, while 42% of these cells expressed both CD133 and CD117. The same results (70–75%) were obtained using CD133 microbeads. As CD133 and CD117 are recognized markers of pro-stem cells, their presence tends to favour the idea that OVCAR-3 cells carry pro-stem cell characteristics.

Due to the crucial role of PAR-1 in EPCR function, we tested the presence of this protein in OVCAR cells. As shown in Fig. 3B, these cells express the PAR-1 protein. This finding was also confirmed by flow cytometry (data not shown).

#### Expression of EPCR by the OVCAR-3 cancer cell line

EPCR expression in OVCAR-3 cells was evaluated. RT-PCR (Fig. 4A) and FACS analysis (Fig. 4B) indicated that both EPCR mRNAs and proteins were detectable in these cells. Other cell lines, derived from lung (A549), colorectal (HT-29) and ileocecal (HCT-8R) cancers, were also screened for EPCR transcription by RT-PCR amplification. Analysis of the product of RT-PCR by gel electrophoresis exhibited a prominent amplification band for all three cell lines. The band size was consistent with the predicted size for EPCR (Fig. 4A).

The integrity of the coding sequences and the EPCR gene locus was further scrutinized through sequencing of the EPCR cDNA and the EPCR gene. The sequence obtained was compared to a consensus sequence corresponding to the endothelial gene. It was observed that the cancer cell and endothelial cell EPCR haplotypes share similarity with respect to single nucleotide polymorphisms (SNPs). For this purpose, both genomic and cDNA were sequenced for OVCAR and MDA-MB231 cell lines, whereas only cDNA was sequenced for A549, HCT-8R and HT-29 cells. The cDNA and genomic DNA sequences were compared with BC01445 (1,381 bp) and AF106202 (8,167 bp) GenBank loci, respectively (Fig. 4). We detected the 13 SNPs already described in endothelial cell genes (Saposnik *et al*) (11). In addition, within the 5′UTR region, a thymidine insertion (locus 260) and an adenosine deletion (locus 840) were found in both MDA-MB231 and OVCAR cells. Moreover, a SNP was observed between the second and the third exon for both cell lines (C5727T). Two other SNPs (G7965C and T8153C) were detected only in the OVCAR cells within the 3′UTR region.

OVCAR, a human ovarian adenocarcinoma cell line carries both C and T at the nucleotide position 3787 and both A and G at nucleotide position 6936 (Fig. 4C). This reflects a heterozygosity for A1 and A3 haplotypes. In the same manner, the breast cancer cell line, MDA-MB231, is heterozygous for A2 and A3 since only C is detected at position 3787, which is located in intron 1, while both A and G are found at position 6936.

The data in Fig. 4D show that the lung cancer cell line, A549, is homozygous for the A3 haplotype, since exon 4 contains G nucleotides at loci 738 and 817. Similar analysis revealed that both colorectal and ileocecal cancer cell lines (HT-29 and HCT-8R) carry the A2 haplotype in the homozygous form.

It is interesting to note that A3 carriers were found to be associated with a large amount of sEPCR shed into their culture medium. It was observed that one million A3 homozygous A549 cells shed approximately 4,250 ng/ml of sEPCR, whereas A3 heterozygous MDA-MB231 and OVCAR cells shed only 450 and 145 ng/ml per million cells, respectively (data not shown).

## Discussion

Data generated recently in our laboratory encouraged us to investigate the role of aPC/sEPCR in ovarian cancers. In the present study, we adressed the question of sEPCR and its levels of expression in ovarian and other cancers, as well as in established cancer cell lines. Our aim was to find out whether sEPCR could be a candidate biomarker.

Our data provide evidence of the presence of EPCR in ovarian cancer cells and also in a large cohort of tumor biopsies. The release of sEPCR is significant since its secretion in the plasma of patients with ovarian cancer is related to enhanced cell survival, invasion and immune down regulation. However, the mechanisms behind this remain to be elucidated. It is of vital importance, in routine clinical practice, to avail of reliable markers of tumor behaviour. CA125 is the currently used marker for ovarian tumors. An additional biomarker of tumor cells, if revealed, would contribute towards making a sounder diagnosis.

EPCR proteins were detected *in situ*, by antibodies against the synthetic peptides of EPCR. In ovarian cancer (90.47%), in the breast (60%), lung (80%) and colon (65%) cancer biopsies. The relatively low percentages of positive samples observed could be an underestimate as TMA samples may contain, besides tumor cells, necrosed tissue or the biopsy may simply miss the tumor nodule and aspirate adipose and/or tumor adjacent healthy tissue. However, TMA analysis of ovarian cancers after treatment showed a decrease in EPCR immunostaining.

aPC is a key inhibitor of fibrin formation. Other than hemostastic function, aPC is also known to exert pleiotropic effects, depending on the cell types expressing EPCR. Among others, aPC interferes with the endothelial cell p53 pathway (19,20). It also promotes endothelial cell proliferation through the MAPK and PI3K signaling pathways (20–22). Cancer cells expressing EPCR may therefore benefit from the cytoprotective effect imparted by aPC (23). Scheffer *et al* found a high expression of EPCR in a large panel of tumor cell lines and interpreted this in the light of the role of EPCR in coagulation (15). Beaulieu and Church (16) claimed that aPC increases breast cancer cell invasion and chemotaxis through EPCR and PAR-1. These findings are demonstrative of the importance of EPCR expression in tumor cell behavior (17). Of note, the OVCAR-3 cell line used in the present study was found to express high levels of CD133 and CD117, which are markers characteristic of pre-stem cells. We also noticed an increased expression of PAR-1, which is a known cofactor for the EPCR signaling pathway in cancer cells.

In addition, we found that ovarian cell-associated EPCR is functional. Treatment of cancer cells with aPC induced cell survival that could be inhibited by the neutralizing antibody, anti-peptide 3. The study of ascites from patients showed that EPCR in the soluble form (sEPCR) could be detected on the cancer cell membranes as well as in the ascitic fluid. In the ascitic fluid, membrane-associated EPCR, via aPC, can inhibit fibrin formation and participate in fluid expansion in ovarian cancer, whereas under intravascular conditions, sEPCR, due to its trapping action, may be a leading cause of hypercoagulable state associated with cancer.

RT-PCR analysis detected the presence of EPCR mRNA in ascitic cell clusters of ovarian cancers, OVCAR-3 cells and several other tumor cell lines, such as breast (MDA-MB231), lung (A549) and colorectal (HT-29, HCT-8R). The presence of EPCR-specific mRNA is in line with and confirms our observation by TMA analysis.

The increase in expression levels of EPCR/sEPCR observed by immune antibodies and TMA was further corroborated by examining the sequence integrity of the EPCR gene and its mRNA transcript. This allowed us to characterize the cancer cell haplotypes which reflects one of the originalities of the current study. EPCR gene sequencing provided evidence for A3 haplotype expression in heterozygous (OVCAR and MDA-MB231) and homozygous (A549) forms. Of note, among the 10 alleles sequenced, 4 represented the A3 haplotype, which is a non-negligible figure. Knowing the frequency in the healthy population (7%), it is questionable whether the values we obtained indeed reflect the malignant nature of the cells studied (11). It might be pertinent to compare the haplotypes of tumor and healthy cells of a patient to see whether the genotype changes with malignancy. The A3 haplotype favors the proteolytic cleavage of EPCR and is thus associated with increased plasma levels of sEPCR (11). It is interesting to note that the cultured cells carrying the A3 haplotype shed large amounts of sEPCR into the medium. Elevated sEPCR plasmatic level increases the risk of venous thrombosis (11,24). Thus, cancer cells that express the A3 haplotype may expose patients to a higher risk level. The A3 haplotype and elevated sEPCR, both of which are inter-related, contribute synergistically to the thrombophilic state in ovarian cancer patients (24).

The results from our study present strong evidence in favour of the role of EPCR/sEPCR in tumor cells, whether in tumor biopsies or in established cancer cell lines. There is a clear increase in sEPCR concomitant with the existence of malignant cells.

CA125 is the currently used marker for ovarian tumors. Nevertheless, on the basis of our observations, we propose that the measurement of plasma sEPCR at regular intervals during the remission period in cancer patients, particularly those with cancer of the ovaries, may provide clinically relevant information and perhaps an early signal warranting attention. The measurement of sEPCR and CA125 simultaneously in ovarian cancer patients could go a step further towards providing a sounder diagnosis.

## Figures and Tables

**Table I:** Quantification of EPCR and CA125 in all the patients.

A, group 1		

Patients	EPCR, ng/ml	CA125 J0
1	68	54
2	86	83
3	64	84
4	69	114
5	57	127
6	117	195
7	85	199
8	57	273
9	82	279
10	43	369.5
11	11	553
12	80	637
Median	68.5	197

B, group 2		

Patients	EPCR, ng/ml	CA125 J0

1	146	30.6
2	229	99.4
3	250	101.3
4	170	122
5	150	140
6	143	148
7	162	265
8	130	296.1
9	180	339
10	291	370
11	198	387
12	251	668
13	274	798
14	140	928
15	253	946
16	230	1130
17	234	3620
Median	198	339

Group 1 comprised 12 patients with a sEPCR level below the baseline (68 to 117 ng/ml). Group 2 comprised patients with a sEPCR level noticeably higher than the plasmatic baseline level (140 to 250 ng/ml, n=17).
